# Effects of low-light stress on aquacultural water quality and disease resistance in Nile tilapia

**DOI:** 10.1371/journal.pone.0268114

**Published:** 2022-05-06

**Authors:** Bingliang Qu, Hui Zhao, Ying Chen, Xiangyong Yu

**Affiliations:** 1 College of Chemistry and Environment, Guangdong Ocean University, Zhanjiang, Guangdong, China; 2 Ocean College, South China Agriculture University, Guangzhou, Guangdong, China; Guangzhou University, CHINA

## Abstract

Light intensity has an important environmental influence on the quality and yield of aquatic products. It is essential to understand the effects of light intensity on water quality and fish metabolism before large-scale aquaculture is implemented. In this study, two low-intensity light levels, 0 lx and 100 lx, were used to stress Nile tilapia (*Oreochromis niloticus*), with a natural light level (500 lx) used as control. The pH, dissolved oxygen and ammonia contents were significantly lower in the water used in the 0 lx and 100 lx groups than in controls, while the levels of nitrite and total phosphorus were apparently higher. Moreover, the numbers of heterotrophic bacteria, *Vibrio* and total coliforms in aquaculture water were 157.1%, 314.2% and 502.4% higher, respectively, after 0 lx light stress for 15 days. The survival rate of Nile tilapia decreased significantly to 90.6% under 0 lx light on the 15^th^ day. Of the immune-related genes, the expressions of IFN-γ, IL-12 and IL-4 were 390.3%, 757.8% and 387.5% higher under 0 lx light and 303.3%, 471.2% and 289.7% higher under 100 lx light, respectively. These results indicate that low-intensity light changes the physicochemical parameters of aquaculture water and increases the number of bacteria it hosts while decreasing the survival rate and increasing the disease resistance of Nile tilapia.

## 1. Introduction

Fish are a crucial source of proteins, vitamins and minerals for humans. Due to rapid declines in catch rates, global aquaculture is swiftly expanding to meet the huge demand for fish [1]. However, substandard practices and hostile aquacultural environments involved in fish rearing have resulted in a range of fish diseases and serious declines in product quality. Hence, it is essential to determine the environmental influences on cultured fish before they are used in large-scale aquaculture.

Light intensity is an important environmental factor in aquaculture. Drastic light intensity alterations in water bodies can generate serious water quality problems [2]. A suitable light intensity degrades pollutants, improves water quality, and eliminates the formation of disinfection byproducts [3]. Proper light conditions are also favorable for the survival [4], growth performance [4], husbandry performance [5], and disease resistance [6] of cultured fishes.

The Nile tilapia (*Oreochromis niloticus*) is known as the “food fish of the 21^st^ century” and is the second most reared fish after carp species [1]. It is widely cultured around the world as it grows rapidly and is hardy, even in hostile aquatic environments [7]. The Nile tilapia is also considered to be a good model for evaluating the influences of environmental factors on cultured fish, because of its strong environmental adaptability. However, few studies have reported the effects of light intensity on the metabolism of Nile tilapia and its aquatic environment.

In this study, three intensities of light were designed to stress Nile tilapia. The quality of their aquatic water body was analyzed in terms of water quality parameters and bacterial content. The disease resistance of fish was analyzed by detecting the survival rate and expression of immune-related genes. The aim was to elaborate upon the effects of low light stress on aquacultural water quality and disease resistance in Nile tilapia.

## 2. Materials and methods

### 2.1 Fish and culturing

Nile tilapia (mean weight = 200 ± 10 g) were bought from an aquaculture farm in Beihai. They were temporarily cultivated in nine 8 m^3^ seawater tanks for 1 week, with 50 fish in each tank. The tanks were randomly divided into three light treatment groups, with three replicate tanks per group. The fish were fed using a basal diet (Yuehai, Zhanjiang, China) twice per day. One-third of the water was changed every 3 days, before which water samples and fish were collected for experiments. The room temperature was controlled by an air conditioner at 28 ± 0.3°C. The experiment was approved by the Animal Ethics Committee of Guangdong Ocean University (protocol number 20190003). All procedures were performed in accordance with the relevant policies of Animal Welfare in China.

### 2.2 Light stress and survival rate analysis

Three light intensities (0 lx, 100 lx and 500 lx) were used to stress the Nile tilapia, of which the 500 lx group was used as controls because 500 lx is close to the optimal light intensity. Daylight lamps (OPPLE, Zhongshan, China) and black nylon nets were used to regulate light intensity. Light intensity was measured at the water’s surface by an underwater irradiance meter (ZDS-10W2D, Shanghai, China). The photoperiod was 12 h light to 12 h dark. The experiment was sustained for 15 days. The survival rates of fish in the three light treatment groups were measured every 3 days.

### 2.3 Determination of water quality parameters

Water samples were collected at 40 cm under the water surface of each tank. The temperature and pH were detected using a thermometer and pH probe (HI2210, Hanna, Italy). The amounts of ammonia, nitrite, total phosphorus, and dissolved oxygen (DO) and the water’s transparency were detected with a water quality testing meter (SJ-7008Ⅱ, SuiJing, China) using the methods described by Gorlach-Lira et al. [8].

### 2.4 Microbiological analyses of water samples

For microbiological analyses, 10 μL water samples were collected from the surface of each tank. They were diluted with 990 μL of 0.9% sterile saline solution and plated on selective media. For heterotrophic bacteria detection, the samples were plated on 2216E medium, including 5 g tryptone, 1 g yeast extract, 0.1 g ferric phosphate, and 20 g agar in 1000 mL seawater (pH 7.6), and cultivated for 3 days at 25°C. For *Vibrio* detection, the samples were done on TCBS medium, including 10 g tryptone, 5 g yeast extract, 10 g NaCl, 10 g Na-citrate, 10 g sodium thiosulfate, 3 g sodium cholate, 5 g Ox-gall powder, 20 g sucrose, 1 g ferric citrate, 15 g agar, 0.04 g bromothymol orchid and 0.04 g thymol orchid in 1000 mL water (pH 8.6), and cultivated for 24 h at 28°C. For total coliforms, samples were cultivated in Lactose Bile 2% Brilliant Green Broth media, which included 10 g enzymatic digest of gelatin, 10 g lactose, 20 g ox bile, and 0.0133 g brilliant green in 1000 mL seawater (pH 7.4) for 5 days at 30°C. The number of total bacteria was counted after cultivation and was represented in terms of the most probable number/100 mL (MPN/100 mL).

### 2.5 RNA isolation, cDNA synthesis and quantitative real-time PCR (qRT-PCR) analysis

Total RNA was extracted from the liver of three experimental fish using RNeasyMini Kits (Qiagen, Gaithersburg, MD, USA). Single-strand cDNA was synthesized from RNA with M-MLV reverse transcriptase (Promega, Madison, WI, USA) and random primer. The qRT-PCR assays were performed with Thermo Scientific DyNAmo Flash SYBR Green qPCR Kits (Thermo Scientific, Waltham, MA, USA). β-actin was used as the internal control. All primers were listed in Table 1. The 2^−△△CT^ method was employed to calculate the relative gene expression levels. Each sample was run in triplicate.

**Table 1:** Primers used in the qRT-PCR analyses.

Primer	Sequence (5’-3’)
IFN-γ-F	AGCGGCTGACTGAACTCAATTGAAG
IFN-γ-R	GTCACAGTTTTCAGCTGTATAGGG
IL-12-F	GGAAGCACGGCAGCAGAATA
IL-12-R	AACTTGAGGGAGAAGTAGGAATGG
IL-4-F	TGTTACCTAAATCTGTTGATCCAG
IL-4-R	TCTGTGGTGTTCTTCGTTGC
β-actin-F	CCACACAGTGCCCATCTACGA
β-actin-R	CCACGCTCTGTCAGGATCTTCA

### 2.6 Statistical analysis

The parameters of each group are expressed as means and standard errors. ANOVA was performed using SPSS 19.0 (IBM, Armonk, NY, USA) to detect significant differences between groups.

## 3. Results

### 3.1 Effect of low-intensity light stress on water quality

The physicochemical parameters of the water samples were presented in Table 2. There were no significant differences in temperature and salinity among all light treatment groups. The mean pHs of the water in the 0 lx, 100 lx and 500 lx groups were 7.5, 7.7 and 8.1, the mean DO values were 5.8 mg/L, 7.3 mg/L and 9.1 mg/L, and the mean ammonia contents were 0.14 mg/L, 0.23 mg/L and 0.26 mg/L, respectively. This indicates that low light reduced the pH, DO and ammonia content of the water. The levels of nitrite, total phosphorus and transparency were 0.008 mg/L, 0.31 mg/L and 23.81 cm under 0 lx, respectively, and reached their minimum values under 500 lx, at 0.003 mg/L, 0.19 mg/L and 21.34 cm. This indicated that low-intensity light upregulated the levels of nitrite, total phosphorus and transparency degree in aquaculture.

**Table 2:** Water quality parameters of the three light treatment groups after 15 consecutive days (mean ± SE and range of variation of 3 tanks).

Parameters	Light density		
	0 lx	100 lx	500 lx
Temperature (°C)	28.04 ± 0.3 (27.8–28.3)	28.14 ± 0.3 (27.7–28.4)	28.38 ± 0.4 (27.6–28.8)
Salinity	19.60 ± 0.4 (19.0–20.1)	19.51 ± 0.3 (19.1–20.1)	19.70 ± 0.4 (19.1–20.2)
pH	7.52 ± 0.5^b^ (7.2–8.2)	7.71 ± 0.3^b^ (7.4–8.1)	8.10 ± 0.6^a^ (7.8–8.4)
Dissolved oxygen (mg/L)	5.81 ± 1.8^b^ (4.9–8.5)	7.32 ± 2.3^ab^ (5.8–11.2)	9.12 ± 3.3^a^ (6.5–12.3)
Ammonia (mg/L)	0.14 ± 0.04^b^ (0.13–0.17)	0.23 ± 0.05^a^ (0.18–0.25)	0.26 ± 0.05^a^ (0.22–0.29)
Nitrite (mg/L)	0.008 ± 0.001^b^ (0.007–0.010)	0.005 ± 0.00 ^ab^ (0.004–-0.007)	0.003 ± 0.001^a^ (0.002–0.005)
Total phosphorus (mg/L)	0.31 ± 0.07^b^ (0.29–0.42)	0.25 ± 0.05^a^ (0.18–0.32)	0.19 ± 0.05^a^ (0.180–0.23)
Transparent degree (cm)	23.81 ± 1.1^b^ (23.1–24.8)	22.12 ± 1.3^a^ (20.1–23.8)	21.34 ± 1.6^a^ (20.4–21.8)

Note: Superscripts with different letters (^a, b, c^) within a row indicated significant differences (*P* < 0.05).

### 3.2 Low-intensity light stress increased bacterial growth in aquaculture water

**Fig 1:**
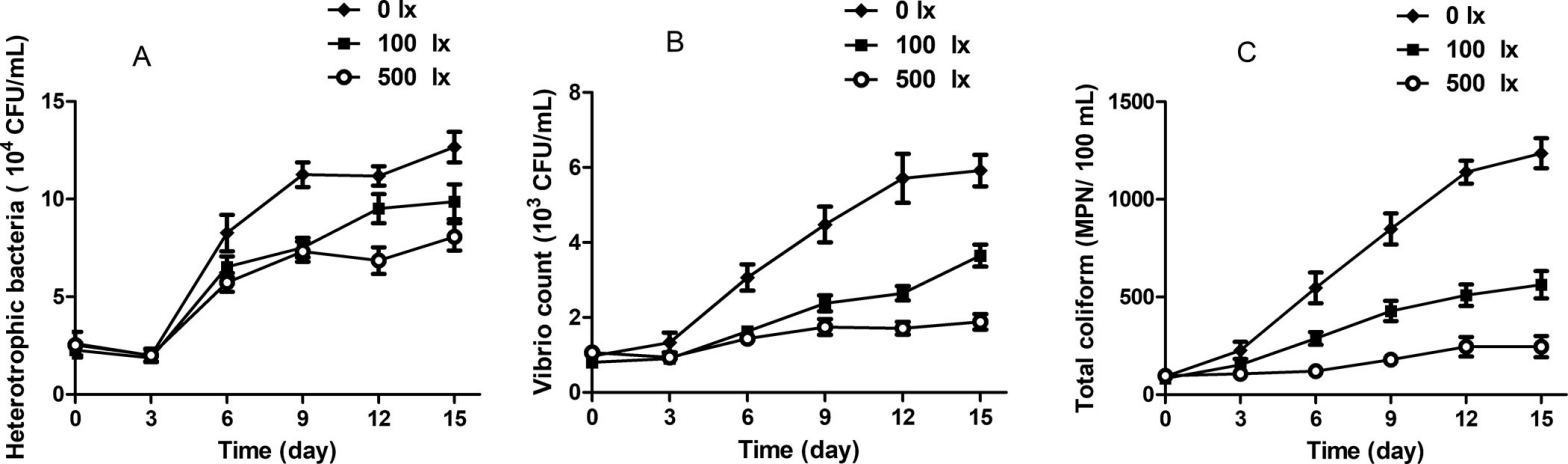
Bacterial growth in aquaculture water under 0 lx, 100 lx and 500 lx illumination: A) heterotrophic bacteria, B) *Vibrio* and C) total coliforms.

To examine the effect of low-intensity light stress on bacteria in water, the numbers of heterotrophic bacteria, *Vibrio* and total coliforms were counted. The numbers of all detected bacteria were increased after fish farming for 15 days. Moreover, there was a significant difference among the three treatment groups on the 15^th^ day. Compared with the 500 lx group (control), the numbers of total heterotrophic bacteria increased by 157.1% in the 0 lx group and 122.3% in the 100 lx group (Fig 1A). Similarly, *Vibrio* numbers were 314.2% and 193.8% higher in the 0 lx and 100 lx groups, respectively (Fig 1B). The total coliform numbers showed more significant changes, increasing by 502.4% (1236 MPN/100 mL) in the 0 lx group and 147.5% (363 MPN/100 mL) in the 100 lx group compared with controls (246 MPN/100 mL; Fig 1C). This indicated that low-intensity light was beneficial to the growth of heterotrophic bacteria, *Vibrio* and total coliforms in the aquaculture water.

### 3.3 Low-intensity light stress decreased the survival rate of Nile tilapia

**Fig 2:**
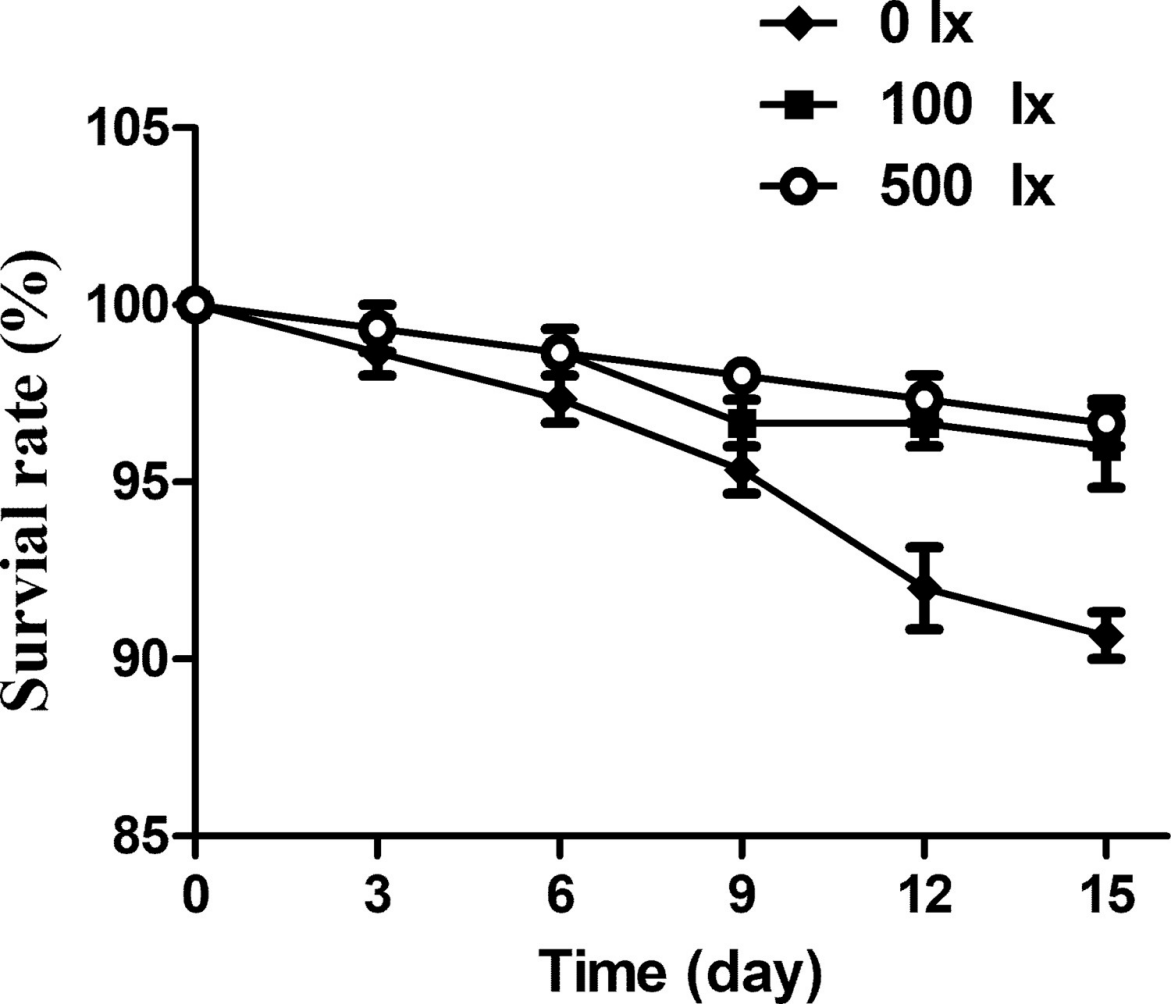
Survival rates of fish under different light levels on the 0^th^, 3^rd^, 6^th^, 9^th^, 12^th^ and 15^th^ days of treatment. Each bar represented the mean of 50 fish.

To investigate whether low-intensity light stress produced serious adverse effects on Nile tilapia, their survival rates were examined on the 0^th^, 3^rd^, 6^th^, 9^th^, 12^th^ and 15^th^ days. As shown in Table 3 and Fig 2, there were no significant differences between the 100 lx and 500 lx groups during the 15-day light treatment (*P* > 0.05). However, in the 0 lx group, the survival rate decreased significantly to 92.7% on the 12^th^ day and declined further to 90.6% on the 15^th^ day compared to controls. This indicates that extreme low-intensity light stress inhibited survival.

**Table 3:** Numbers of deaths of Nile tilapia every 3 days under different light levels.

Light intensity (lx)	Death number (150 fish per treatment group)						
	0 day	3^th^ day	6^th^ day	9^th^ day	12^th^ day	15^th^ day	Sum
0	0	2	2	3	4	3	14
100	0	1	1	3	0	1	6
500	0	1	0	1	1	1	4

### 3.4 Low-intensity light stress activated the expression of immune-related genes in Nile tilapia

**Fig 3:**
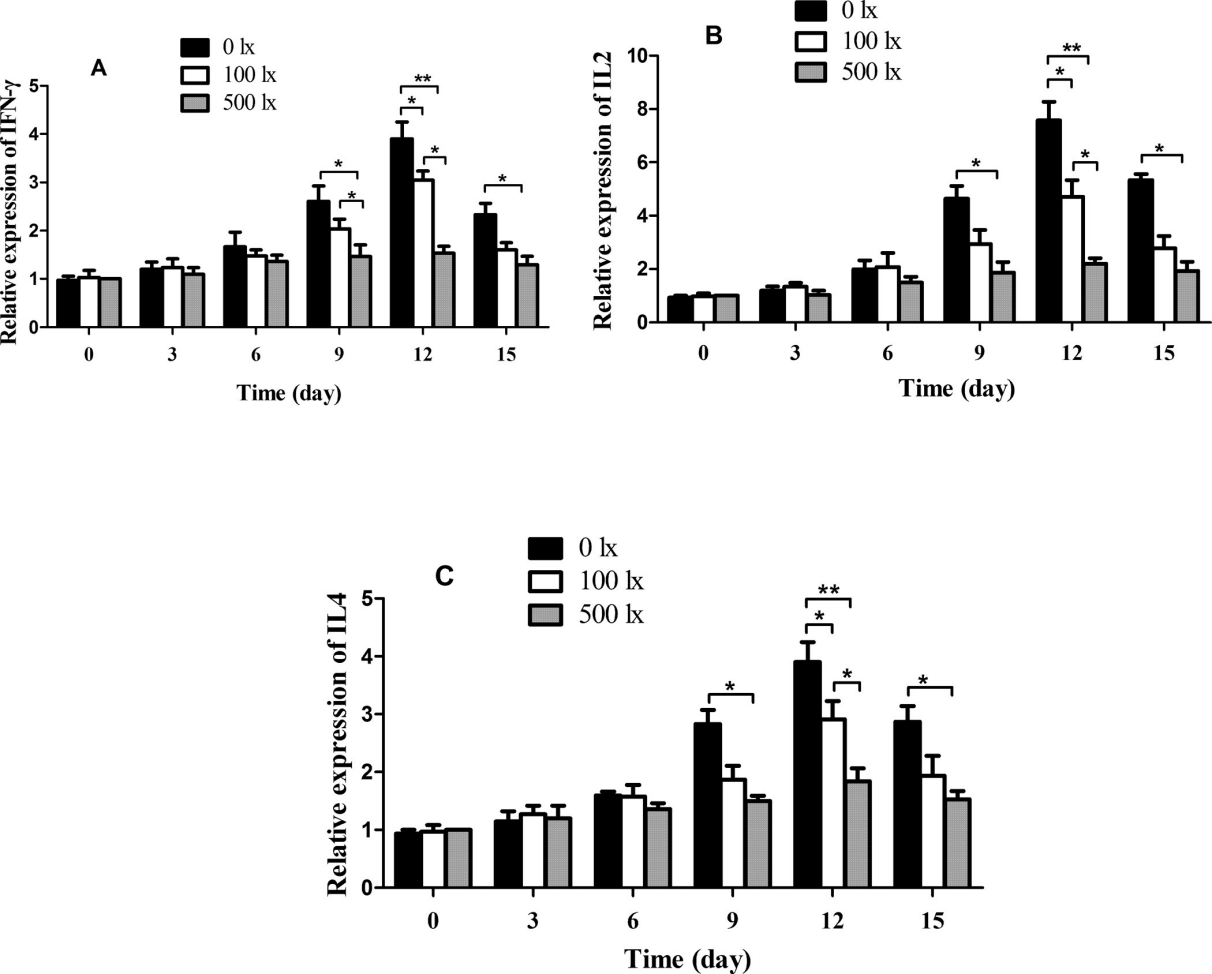
Expressions of A) IFN-γ, B) IL-12 and C) IL-4 in the liver of Nile tilapia under three light levels according to qRT-PCR. β-actin was run as the control. Data are expressed as mean ± SE (*n* = 3). *P < 0.05 and **P < 0.01.

Because low-intensity light decreased the survival rate of Nile tilapia, it was speculated that it acted as a stressor that stimulated the immune response. Hence, the expressions of interferon gamma (IFN-γ), interleukin 12 (IL-12) and interleukin (IL-4) were analyzed in each group (Fig 3). In the 0 lx group, the expressions of IFN-γ, IL-12 and IL-4 did not change during the first 8 days (*P* > 0.05), but were significantly up-regulated on the 9^th^ day (*P* < 0.05) and reached 390.3%, 757.8% and 387.5% of their initial values, respectively, on the 12^th^ day (*P* < 0.01), before being down-regulated afterward (*P* < 0.05). The 100 lx group showed lesser changes. The IFN-γ, IL-12 and IL-4 transcripts were increased by 303.3%, 471.2% and 289.7%, respectively, on the 12^th^ day (*P* < 0.05) and recovered to normal levels subsequently (*P* > 0.05). This indicated that low-intensity light activated the immune response of Nile tilapia.

## 4. Discussion

Light intensity is a crucial environmental factor that influences aquacultural water quality, such as temperature, pH, and levels of DO, ammonia, nitrite and total phosphorus [9, 10]. In this research, as expected, low-intensity light significantly down-regulated the pH, DO and ammonia content of aquaculture water, but up-regulated the levels of nitrite and total phosphorus. This was speculated to be due to weak photosynthesis occurring under low-intensity light [11], which generated less O_2_ and decreased DO. While strong respiration by fish generated CO_2_ and hydrogen ions, which decreased pH [12, 13]. Meanwhile, due to the lack of O_2_, nitrate did not readily reduce to ammonia, so there were high nitrate and low ammonia contents [14]. In addition, weak photosynthesis inhibited algal growth and increased water transparency. Fewer algae reduced the need for phosphorus, so its content increased under low-intensity light [15, 16].

Light is considered the single most important contributor to bacterial die-off in aquaculture water [17, 18]. A bactericidal effect of light was achieved that caused a rapid decrease in the colony-forming ability of bacteria [19, 20]. Solar UV light is highly bactericidal, causing direct photobiological DNA damage in bacteria [21]. In this study, the numbers of all detected bacteria were very low under 500 lx light and were significantly higher under 0 lx and 100 lx light. This demonstrated that low-intensity light results in the rapid proliferation of bacteria in aquaculture water.

Changes in the physicochemical properties of aquaculture water, such as pH, directly influence bacterial proliferation [8, 22]. Seawater pH normally ranges from 7.5 to 8.5 and is influenced by light, temperature, pressure and the respiratory activities of microorganisms [23]. It has been reported that a pH of approximately 8 has the strongest deleterious effects on *Vibrio* and total coliforms in seawater [18]. In this report, the numbers of all detected bacteria were lowest in the 500 lx group, in which the pH was close to 8.0 at 8.1. Meanwhile, the numbers of bacteria were significantly higher in the 0 lx and 100 lx groups, which had pHs of 7.5 and 7.7, respectively. The data suggest that low pH due to low-intensity light allowed high bacterial survival. Similar results were reported by Joux et al. [24], who showed that an acidic pH favored the survival of total coliforms in both seawater and NaCl solution, and that survival decreased with the increase in pH.

Light can directly affect the survival, growth, swimming, aggression, hatching, metabolism and immune response of fish [6]. According to previous research, insufficient light leaded to poor growth and high mortality in fish [25]. In the present report, the extremely low light treatment (0 lx) decreased the survival rate of fish to 90.6%, significantly lower than that of controls. However, there was no obvious difference in survival rate between the 100 lx and control groups. A reasonable explanation is that Nile tilapia are visual predators and need a minimum light intensity to feed and grow normally [6]. Complete darkness led to abrupt changes in the rearing conditions and growth environment, which decreased survival.

Visible light exposure can modulate immune function [26, 27]. Although fish initiate immune responses through a variety of signal recognition and signal transduction pathways, inflammatory cytokines play important antiviral or antibacterial roles in the last step [28, 29]. IL4, IFN-γ and IL12 are inflammatory cytokines that are widely studied in relation to immune response. It has been reported that IL4 can enhance IFN-γ expression in murine NK cells in coordination with the T1 cytokine IL12 [30, 31], implying that IL4, IFN-γ and IL12 might have been working together in the present study. In addition, transcriptome analysis of samples from the three treatment groups showed that IL4, IFN-γ and IL12 were significantly differently expressed (data not shown). Therefore, IL4, IFN-γ and IL12 were selected for analysis in this research. After low-intensity light stress, the expression levels of IFN-γ, IL-12 and IL-4 were significantly up-regulated, which were consistent with the changes in bacterial numbers in the aquaculture water. This consistency indicated that the high bacterial content resulting from low-intensity light stress might be the main influence on the immune response of fish. However, the gene expression levels gradually recovered after the 12^th^ day, suggesting that the fish might have adapted to the low-intensity light environment by inhibiting the transcription of certain immune-related genes.

## 5. Conclusion

This study demonstrated the effects of different light intensities (0 lx, 100 lx and 500 lx) on aquaculture water quality and disease resistance in Nile tilapia. The pH, DO and ammonia content of aquaculture water were significantly lower in the 0 lx (no light) and 100 lx groups (reduced light) than in the 500 lx group (control, representing the natural light level). The levels of nitrite and total phosphorus were apparently higher in the 0 lx and 100 lx groups than in the 500 lx group. Moreover, the 0 lx group had significantly higher numbers of heterotrophic bacteria, *Vibrio* and total coliforms and a significantly decreased Nile tilapia survival rate. The expressions of immune-related genes, including IFN-γ, IL-12 and IL-4, were significantly higher in the 0 lx and 100 lx groups. These results indicate that low-intensity light changes the physicochemical parameters of aquaculture water and up-regulates the number of bacteria. Meanwhile, low-intensity light decreases the survival rate and stimulates disease resistance in Nile tilapia.
